# 

**Published:** 1874-08

**Authors:** 


### CONTENTS.

### COMMUNICATIONS:

Conservative Treatment of the Dental Pulp ........... 317
Report on Dental Education ........................ 325
Dental Ethics ....................................... 331
Cheap Polishing Wheels and Cones ................. 336
PROCEEDINGS OF SOCITIES:
The District Dental Society of the 7th Judicial District of the State of N. Y. ............................ 336
Mad River Valley Dental Society .................. 338
The Sixth Annual Meeting of the Dental Society of the State of N. Y. .................................. 339
Northern Ohio Dental Association ................. 340
Proceedings of Tenn. Dental Association, Eighth Annual Meeting ............................................ 345
CORRESPONDENCE:
EDITORIAL:
Obituary ............................................ 359
Resolutions of Respect adopted by the Dentists of Nashville to the late Dr. Hamlin ......................... 360

